# Alleviation of Neurological Disorders by Targeting Neurodegenerative-Associated Enzymes: Natural and Synthetic Molecules

**DOI:** 10.3390/ijms26104707

**Published:** 2025-05-14

**Authors:** Alka Ashok Singh, Fazlurrahman Khan, Minseok Song

**Affiliations:** 1 Department of Life Sciences, Yeungnam University, Gyeongsan 38541, Republic of Korea; alkasingh10f@yu.ac.kr; 2 Ocean and Fisheries Development International Cooperation Institute, Pukyong National University, Busan 48513, Republic of Korea; 3 International Graduate Program of Fisheries Science, Pukyong National University, Busan 48513, Republic of Korea

**Keywords:** neurodegenerative diseases, enzyme inhibition, natural molecules, synthetic molecules, mechanisms of action, acetylcholinesterase, monoamine oxidase, beta-secretase, tau kinases, caspases, cyclooxygenase-2

## Abstract

Neurological disorders, encompassing neurodegenerative and neuroinflammatory conditions, present significant public health and clinical challenges. Recent research has elucidated the pivotal role of various enzymes in the onset and progression of these disorders. This review explores the therapeutic potential of targeting these enzymes with natural and synthetic molecules. Key enzymes, including acetylcholinesterase, monoamine oxidase, beta-secretase, tau kinases, caspases, and cyclooxygenase-2, are implicated in diseases such as Alzheimer’s disease, Parkinson’s disease, and multiple sclerosis. Modulating these enzymes can alleviate symptoms, slow disease progression, or reverse pathological changes. Natural molecules derived from plants, microbes, seaweeds, and animals have long been noted for their therapeutic potential. Their ability to interact with specific enzymes with high specificity and minimal side effects makes them promising candidates for treatment. These natural agents provide a foundation for developing targeted therapies with improved safety profiles. Simultaneously, the development of synthetic chemistry has resulted in molecules designed to inhibit neurodegenerative enzymes with precision. This review examines the progress in creating small molecules, peptides, and enzyme inhibitors through sophisticated drug design techniques. It evaluates the efficacy, safety, and mechanisms of these synthetic agents, highlighting their potential for clinical application. The review offers a comprehensive overview of recent advancements in enzyme-targeted therapies for neurological disorders, covering both natural and synthetic molecules investigated in preclinical and clinical settings. It discusses the mechanisms through which these molecules exert their effects, the challenges faced in their development, and future research directions. By synthesizing current knowledge, this paper aims to illuminate the potential of enzyme-targeted interventions in managing neurological disorders, showcasing both the promise and limitations of these approaches.

## 1. Introduction

Neurological disorders, including neurodegenerative and neuroinflammatory conditions, present significant challenges to global health [1]. These disorders are characterized by chronic neuroinflammation, which contributes to disease progression [2,3]. An effective treatment for neurodegenerative disorders remains elusive, highlighting the need for continued research and innovative approaches to address this growing public health concern [2,3]. Neurodegenerative diseases, including Alzheimer’s, Parkinson’s, and Huntington’s, as well as neuroinflammatory conditions like multiple sclerosis, are characterized by progressive neuronal damage and dysfunction [2,4]. Neuroinflammation, characterized by glial cell activation and release of pro-inflammatory mediators, is a unifying mechanism in many neurological disorders [5,6]. This chronic immune activation in the central nervous system is responsible for disease progression and may even initiate neurodegenerative processes [7,8].

Recent research has highlighted the crucial role of enzymes in neurological disorders, encompassing Alzheimer’s, Parkinson’s, and Huntington’s diseases. Enzymes contribute to disease pathology by influencing neurotransmitter metabolism, protein aggregation, and neuroinflammation [9,10]. Targeting these enzymes has emerged as a promising therapeutic strategy, with enzyme inhibition showing potential in slowing disease progression [11]. Several key enzymes, such as acetylcholinesterase (AChE), beta-secretase (*BACE1*), tau kinases, monoamine oxidase, caspases, and cyclooxygenase-2, reportedly play significant roles in the development of neurological disorders [12,13].

AChE plays a crucial role in the pathogenesis of Alzheimer’s disease (AD) by influencing inflammation, apoptosis, oxidative stress, and protein aggregation [14]. AChE inhibitors are used to treat AD symptoms by increasing acetylcholine levels [15]. However, AChE may also interact directly with amyloid-beta, promoting plaque formation [15,16].

Monoamine oxidase (MAO) plays a crucial role in neurotransmitter metabolism, particularly dopamine, serotonin, and norepinephrine [17]. MAO inhibitors have been developed to address neurotransmitter imbalances in Parkinson’s disease (PD) and other neurological disorders [18,19]. While initially believed to primarily degrade dopamine, recent research suggests that MAOB in astrocytes may contribute to PD pathology through GABA and hydrogen peroxide production [20]. MAO-generated hydrogen peroxide can impair mitochondrial function, linking MAO activity to mitochondrial defects observed in PD [21].

Caspases, a family of proteases involved in cell death and inflammation, are essential to the pathophysiology of AD [22]. These enzymes contribute to amyloid-β processing, tau cleavage, and neuroinflammation [23,24]. Caspase activation has been observed in AD brains, with caspase-cleaved tau potentially accelerating tangle formation [23,25]. Significantly, caspases are involved in microglial activation, a key process in neurodegenerative disorders [26].

Cyclooxygenases, lipoxygenases, and epoxygenases are implicated in neuroinflammation and oxidative stress associated with neurological disorders [27]. Cyclooxygenase-2 (COX-2) plays a complex role in neuroinflammation and neurodegenerative disorders, including multiple sclerosis (MS). While traditionally considered pro-inflammatory, COX-2 can mediate neuroprotection and contribute to normal brain functions [28,29]. COX-2 is involved in synaptic signaling and plasticity through its production of prostaglandins and metabolism of endocannabinoids [30]. In MS lesions, COX-2 expression is markedly induced and associated with recent demyelination [31]. The arachidonic acid pathway, including COX-2, contributes to MS pathology and symptoms [32,33]. However, recent evidence suggests that COX-1 may play a more significant role in neuroinflammation than previously thought [34]. Metalloenzymes have also been identified as attractive targets for therapeutic intervention in various diseases [35].

Traditional approaches targeting these enzymes individually have shown limited efficacy, increasing interest in multi-target directed ligands (MTDLs) [36,37]. Natural compounds and synthetic molecules have demonstrated the potential to modulate multiple AD-related enzymes simultaneously [38,39]. Recent research has focused on developing novel inhibitors and enhancers targeting these enzymes and exploring new therapeutic strategies, such as autophagy and synaptogenesis [40,41].

Natural compounds produced from plants, in particular polyphenols, alkaloids, and flavonoids, have demonstrated a considerable neuroprotective potential against several different neurodegenerative diseases (NDDs), including HD, PD, and AD [42,43]. Alkaloids such as isoquinoline, indole, and piperidine derivatives act as adenosine receptor agonists, muscarinic, MAO inhibitors, and acetylcholinesterase inhibitors [44,45]. Similarly, compounds such as resveratrol, curcumin, quercetin, and epigallocatechin-3-gallate (EGCG) [46] have all shown neuroprotective effects by modulating enzyme activity, reducing oxidative stress, and mitigating disease-related changes in preclinical models [38,47]. These natural compounds often interact with specific enzymes, including acetylcholinesterase and β-secretase, with high selectivity and minimal side effects [48]. Their ability to restore efficient autophagy and dismantle misfolded proteins contributes to their neuroprotective properties [49]. The inherent safety profile of these compounds, coupled with their multifaceted mechanisms of action, provides a foundation for developing more targeted and less toxic therapies for neurodegenerative disorders [50].

Recent advances in synthetic chemistry have led to the development of novel molecules targeting neurodegenerative diseases. These include small molecules, peptides, and enzyme inhibitors designed to interact specifically with target enzymes [51,52]. Multi-targeted designed drugs (MTDDs) show promise in preclinical studies, utilizing scaffolds like polycyclic cage compounds and thiazolidinediones [52]. Synthetic biology approaches are employed to design gene networks and vectors targeting disease-related genes [53]. Researchers focus on modulating protein aggregation processes common in neurodegenerative diseases, developing peptide-based agents, and multi-target-directed ligands [54]. *BACE1*, a key enzyme in amyloid-β production, is considered a primary therapeutic target [55,56]. Various *BACE1* inhibitors have been developed, ranging from peptidomimetic to non-peptidic compounds, with some demonstrating efficacy in animal models [57,58].However, challenges remain in developing inhibitors with optimal drug-like properties and brain penetration [59]. Additionally, kinase inhibitors have been developed to target tau hyperphosphorylation, particularly focusing on GSK3β, CDK5, and ERK2 [60]. Despite promising preclinical results, many *BACE1* inhibitors have failed in late-stage clinical trials, raising concerns about their safety and efficacy [61]. Despite promising preclinical results, many *BACE1* inhibitors failed in clinical trials due to poor blood–brain barrier penetration, off-target effects, and dose-limiting toxicities [62].

This review was conducted by systematically searching the scientific literature to identify relevant studies addressing specific topics, such as “natural compounds in neurological disorders, mechanisms targeting neurodegenerative enzymes, Neurodegenerative Diseases; Enzyme Inhibition; Natural Molecules; Synthetic Molecules; Mechanisms of Action; Acetylcholinesterase; Monoamine Oxidase; Beta-Secretase; Caspases; Cyclooxygenase-2”. Databases such as PubMed, Scopus, Web of Science, Google Scholar, NCBI, and SCIHUB were searched with combinations of the terms “neurological disorders”, “Alzheimer’s disease”, “Parkinson’s disease”, and “neuroprotection”. This review article is an update not only on the recent advances in the search for credible biomarkers but also on the newer detection techniques and therapeutic approaches targeting neurodegenerative diseases. The review presented herein aims to provide a comprehensive overview of recent advancements in enzyme-targeted therapies for neurological disorders. It will cover both natural and synthetic molecules investigated in preclinical and clinical settings, emphasizing their modes of action, efficiency, and safety profiles.

## 2. Role of Multiple Enzymes Linked in the Progression of Neurological Disorders

There are several enzymes, such as sphingomyelinases, acetylcholinesterase, monoamine oxidase, beta-secretase (*BACE1*), Tau kinases, caspases, and cyclooxygenase-2 (COX-2), which are directly or indirectly linked with the progression of neurological disorders. These enzymes play crucial roles in neurological disorders, particularly Alzheimer’s disease (AD). Sphingomyelinases, which produce ceramide, are implicated in various neurological conditions and represent potential therapeutic targets [63]. AChE inhibitors are among the few approved AD treatments, while other enzymes like secretases, caspases, and sirtuins contribute to AD pathogenesis [41]. Novel dual inhibitors of neutral sphingomyelinase-2 (nSMase2) and AChE have shown promise in suppressing tau pathology propagation in AD models [64]. Cyclooxygenase-2, traditionally viewed as pro-inflammatory, may also have neuroprotective properties. Conversely, COX-1, once considered homeostatic, is actively involved in neuroinflammation induced by various stimuli, including amyloid-beta. This suggests that COX-1 inhibition could be a viable therapeutic approach for neuroinflammatory diseases like AD [29]. The following is a detailed discussion of the enzymes and their role in the progression of neurological disorders.

### 2.1. Sphingomyelinases

Sphingomyelinases, enzymes that catalyze sphingomyelin hydrolysis into phosphorylcholine and ceramide, are highly prevalent in the brain. They serve as an efficient and critical source of ceramide production in normal physiological responses to receptor activation and in the pathogenesis of various neurological disorders [63]. Emerging studies are investigating therapeutic strategies that target sphingomyelinase activity and ceramide synthesis, such as the use of small-molecule inhibitors and gene silencing techniques. They are divided into several subtypes based on pH optimality and cation reliance. Alkaline sphingomyelinase, magnesium-independent N-SMase, magnesium-dependent N-SMase, secreted zinc-dependent acid sphingomyelinase (aSMase), and lysosomal acid sphingomyelinase are some of the subtypes that fall under this category [65]. N-SMase is activated in hippocampal neurons [66], and the ceramide produced by N-SMase is crucial to modulating memory-related synaptic activity. A study shows that rat astrocytes in the hippocampus show elevated ceramide levels and induction of N-SMase activity following brain ischemia. Following cerebral ischemia, N-SMase activity inhibition regulates the rise of ceramide and neuronal damage [67]. Both Ab oligomers and hyperphosphorylated tau have been reported to contribute to neuronal death in this condition. In Alzheimer’s disease, higher levels of aSMase and acid ceramidase reduce sphingomyelin and increase ceramide. Brain aSMase is linked to high levels of Ab or hyperphosphorylated tau [68]. Desipramine, which is an aSMase inhibitor, can reduce the effect of cholesterol oxidation products such as 27-hydroxycholesterol, total lipids from LDL, and oxidized LDL on the production of antibodies in SHSY5Y cells [69]. Patients with Alzheimer’s disease and mouse models have been found to have higher amounts of aSMase in their fibroblasts, brain, and plasma [70]. On the other hand, patients with AD have a lower level of aSMase in their CSF [71].

### 2.2. Acetylcholinesterase (AChE)

AChE is a member of a substantial protein family with a highly conserved structure [72]. After identifying the cholinergic deficiency in AD, AChE has been thoroughly investigated in tissues linked to AD. Additionally, it has been demonstrated that AChE’s peripheral anionic site creates a stable combination with components of the senile plaque. Furthermore, the presence of AChE increases the neurotoxicity of amyloid’s components. The existence of amyloid aggregates in AD is intimately linked to the incidence of altered glycosylation of certain AChE forms [73]. An intriguing investigation on the implications of activity and molecular form alterations in AD discovered that AChE in glycosylated AChE form in the frontal cortex and CSF of Alzheimer’s patients differs from that in non-AD populations, including those with different kinds of dementia [74]. Currently, improving cholinergic neurotransmission remains a primary technique in the symptomatic management of intellectual and behavioral changes in mild to moderate Alzheimer’s disease.

Various compounds, such as linopirdine, a drug that enhances hippocampal acetylcholine (Ach) release, muscarinic ACh receptor agonists such as xanomeline, and AChE inhibitors like physostigmine and tacrine, have been used in this therapeutic method. ACh is hydrolytically degraded in the brain by two cholinesterases: butyrylcholinesterase and AChE [75]. During the progression of Alzheimer’s disease, the activity of AChE in the temporal lobe and the hippocampus decreases by 67% below average levels, whilst the activity of BuChE increases by up to 165% of normal levels. Furthermore, reduced BuChE activity in the medial temporal cortex was associated with gradual cognitive deterioration [76].

AChE inhibitors have been extensively employed to treat AD. These medications restore synaptic levels of ACh and restrict its turnover, compensating for the loss of cholinergic neurons and providing symptomatic relief [15]. AChE can degrade ACh transmission from the presynaptic membrane and inhibit neurotransmitter excitatory effects on the postsynaptic membrane, which is essential for nerve transmission. Acetylcholinesterase inhibitors (AChEIs) can delay the breakdown of ACh, making them an important treatment method for Alzheimer’s disease [77].

Therapeutic interventions. ACh is found throughout the nervous system and has been implicated in controlling numerous brain activities. ACh modulates synaptic transmission and promotes synaptic plasticity, notably in the hippocampus and cortex, and so has a significant part in learning and memory regulation [78]. An enzyme known as AChE is responsible for the degradation of the neurotransmitter ACh at cholinergic synapses. It can be found in the central nervous system as well as the peripheral nervous system. Consequently, it is an important therapeutic target for disorders characterized by cholinergic deficiency, including Alzheimer’s disease [79]. Acetylcholinesterase inhibitors (AChEIs) used to treat Alzheimer’s disease may reduce inflammatory processes that contribute to AMD pathogenesis. AChEIs have been demonstrated to lower pro-inflammatory cytokines linked with NLRP3 activation, as well as neuroinflammation, by inhibiting the NLRP3 inflammasome, NF-kb/STAT3 phosphorylation, and AKT/MAPK signaling [80,81,82,83,84].

### 2.3. Monoamine Oxidase

Monoamine oxidases A and B (MAO A and MAO B) are flavoproteins attached to the outer membrane of the mitochondria that catalyze the oxidative deamination of biogenic amines and neurotransmitters. Many mechanism-based inhibitors (MAOIs) have been created for both neuroprotective and depressive therapeutic applications [85]. MAO-B inhibitors are well tolerated in the treatment of Parkinson’s disease due to their pharmacokinetic features and neuroprotective impact. Rasagiline and selegiline were suggested compounds for early Parkinson’s disease because they are safe and give a small to significant increase in motor function while delaying the introduction of levodopa [86]. In the human brain, MAO-B is insensitive to clorgyline and deaminates PEA and, to a high degree, dopamine. In contrast, MAO-A selectively inhibits at low concentrations (µM) of clorgyline and deaminates serotonin, noradrenaline, and tyramine [87].

Parkinson’s disease (PD) symptoms are frequently treated with monoamine oxidase-B (MAO-B) inhibitors. While MAO-B inhibitors have been used extensively as adjuvant medications to treat PD in its advanced phases, MAO-B inhibitor monotherapy is safe and effective for treating PD in its early stages. In addition to reducing “OFF” time and effectively improving patients’ motor and non-motor symptoms, MAO-B inhibitors could put an end to the disease’s progression [88]. MAO-A inhibitors are particularly useful when treating depression, whereas MAO-B inhibitors are used to treat PD. It is also being studied whether MAO-B inhibitors are beneficial in treating AD [89]. It has been proposed that the recently released MAO-B inhibitor safinamide also inhibits the pathological release of glutamate. Currently, selegiline and rasagiline are used in combination with levodopa and early therapy. Safinamide is only authorized for use in conjunction with levodopa in cases of motor irregularities. An important treatment option for Parkinson’s disease that is all too frequently underappreciated is MAO-B inhibitors [90].

### 2.4. Beta-Secretase (BACE1)

**Figure 1:**
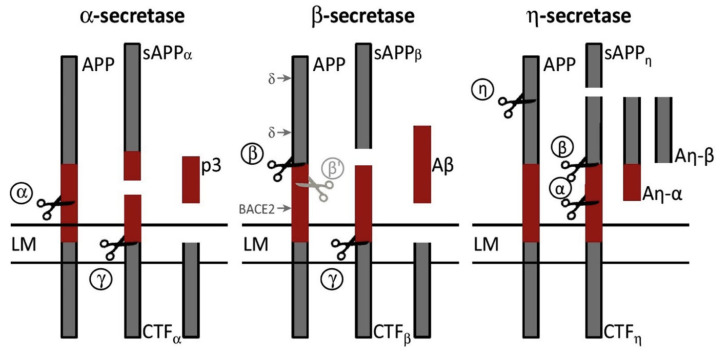
The processing mechanisms for amyloid precursor protein (APP) are represented schematically. Aβ, Aη-α, and Aη-β are abbreviations for amyloid-β, amyloid-η-α, and amyloid-η-β, respectively. Other abbreviations include BACE2, beta-site amyloid precursor protein cleaving enzyme 2, CTF, LM, p3, and soluble amyloid precursor protein (sAPP). Reprinted with permission from [96], Copyright © 2016 Elsevier Ltd.

*BACE1* was first transcribed and described in 1999. Producing all monomeric forms of amyloid-β (Aβ), such as Aβ42, which is thought to be the source of toxicity in AD and aggregates into bioactive conformational species, requires a specific condition. These findings support the idea that *BACE1* has an important role in the etiology of AD [91]. *BACE1* levels and activity rates are elevated in AD brains and body fluids. There is a significant amount of *BACE1* expression in the brain, primarily in neurons, oligodendrocytes, and astrocytes, with increased expression levels in particular neuronal cell types [92,93]. In addition to its presence in the plasma membrane and endosomal compartments, *BACE1* can be identified in healthy synaptic terminals and dystrophic neurites surrounding amyloid-β (Aβ) plaques [94,95]. In Alzheimer’s disease (AD), the β-secretase enzyme *BACE1* cleaves the transmembrane amyloid precursor protein (APP). The APP is also cleaved by α-secretase, which releases *sAPP*α. In conjunction with γ-secretase, it forms Aβ species, which are responsible for creating increasingly extensive and conformationally complex soluble regionally deposited brain aggregates. The cleavage of APP by *BACE1* is the phase responsible for the rate-limiting effect in generating Aβ (Figure 1) [91].

*BACE1*, an aspartic protease, initiates the development of Aβ, a primary component of amyloid plaques, a key pathogenic characteristic of this condition. Thus, targeting *BACE1* for disease-modifying AD treatments is a rational strategy. The aggregate knowledge gained by exploring *BACE1* deletion mutants and characterizing *BACE1* substrates has downstream implications for identifying AD medication treatments and anticipating the adverse effects of *BACE1* inhibitors [97]. The most comprehensive understanding of *BACE1*’s role in myelination is currently available. During the early stages of postnatal development, *BACE1* plays a direct role in the myelination of the peripheral nervous system by cleaving and activating Neuregulin-1. However, little is known about other physiological processes unique to the central nervous system. At least some AD brains have elevated *BACE1* [98]. *BACE1* and γ-secretase are potential targets for treating Alzheimer’s disease. It has been demonstrated that *BACE1* prefers heavier residues at P1, as seen in APPswe [99]. Human brain extracts showed significant levels of *BACE1* enzymatic activity. Neurons produce the most Aβ, consistent with prior studies [100,101]. Recent findings suggest that *BACE1* has a role in the breakdown of Aβ42 in its less toxic and more soluble variants [102,103]. It discovered an increase in *BACE1* activity in MCI-AD patients’ serum, which correlates with the deposition of neurotoxic Aβ prior to the onset of AD symptoms. This finding reveals the high ability of serum *BACE1* activity to identify patients with a high risk of acquiring Alzheimer’s disease at an early stage [104].

### 2.5. Tau Kinases

Tau is an extensively transcribed microtubule-binding protein known to be mostly produced in neurons [105,106,107]. The particular mechanisms and precipitating events that cause aberrant tau accumulation are unknown; however, phosphorylation is thought to play a crucial role [108]. Tau primarily occurs in the human brain in six distinct isoforms, which differ in whether or not the second of four microtubule-binding repeats and one or two N-terminal acidic repeats are present [109]. Tau is a protein that is associated with neuronal microtubules. Its hyperphosphorylation is known to contribute significantly to Alzheimer’s disease. Tau expression and phosphorylation are developmentally regulated, but their dynamic regulation and the kinases or phosphatases that control them remain unknown [110]. Tau is a prominent microtubule-associated protein (MAP) in the brain. Its principal biological role is to increase microtubule assembly and stability. The mature human brain expresses six tau isoforms due to alternative mRNA splicing from a single gene [111]. Phosphorylation generally controls tau’s biological function to negatively encourage microtubule construction, whereas aberrant hyperphosphorylation, similar to AD, causes tau to impede microtubule formation and polymerize into PHFs/NFTs [111,112]. Like PHF-tau, fetal tau is also strongly phosphorylated at numerous sites [113]. Despite this, no evidence suggests that the fetal brain does not exhibit PHF/NFT development or microtubule assembly. A significant number of protein kinases and phosphatases that are responsible for mediating tau phosphorylation have been identified as a result of research into abnormal tau hyperphosphorylation processes in the brain of Alzheimer’s disease patients. For example, GSK-3β, CDK5, MAPKs, PKA, CaMKII, and Dyrk1A are all examples of tau kinases [114]. It should be acknowledged that the C-terminal end of tau comprises the sites Ser396, Ser404, and Ser422, which were only slightly to moderately phosphorylated in the developing brain but were highly phosphorylated in the brain of an Alzheimer’s disease patient. Because of this, a considerable amount of hyperphosphorylation of tau at the C-terminus may be the reason for tau’s inherent tendency to self-aggregate into NFTs. Following the previous findings, the process of phosphorylating tau at its C-terminus in vitro by GSK-3β increases tau self-aggregation [115]. Tau is more fibrillogenic than wild-type tau when its alterations, Ser396, Ser404, and Ser422, change into glutamate to simulate phosphorylation [116,117]. Additionally, the alanine mutation of Ser422 prevents tau from aggregating due to β-amyloid [118]. These studies shed light on the possible sites required for the abnormal hyperphosphorylation of tau in Alzheimer’s disease and provide novel insights into the developmental regulation of site-specific tau phosphorylation.

### 2.6. Caspases

Caspases are a group of protease enzymes that play an important role in apoptosis. Caspase activation dysregulation has been linked to various clinical diseases, making caspases an intriguing target for study into cell death mechanisms and therapeutic methods for diseases related to aberrant apoptosis [119]. Caspases play a crucial role in modulating immunological and inflammatory responses by activating cytokines and signaling pathways associated with cell death. They control programmed cell death (PCD), including apoptosis [120].

It has become increasingly apparent that caspases are significant in the neuroinflammatory reactions and neuronal death in various neurological and neuropsychiatric conditions [121]. These conditions include Alzheimer’s disease (AD), Parkinson’s disease (PD), Huntington’s disease, [121] multiple sclerosis, amyotrophic lateral sclerosis, tauopathies, and age-related macular degeneration [122,123,124]. Recent research has significantly enhanced our understanding of the peculiar functions that caspases play in mediating pain perception, which is an intriguing development. Caspase-1, caspase-3, and caspase-6 are acknowledged as key signaling molecules for the establishment and persistence of nociception [125,126,127] in the spinal dorsal horn [128]. This is because they modulate neuroinflammation, neuronal apoptosis, and synaptic plasticity.

Ischemic stroke was described as the first neurologic disorder in which the activation of a caspase (caspase 1) was detected [129]. Additionally, caspase inhibition prevents tissue damage and enables notable neurologic improvement [130]. Cerebral ischemia has been shown to activate caspases 1, 3, 8, 9, and 11 and release cytochrome c [131,132], and there has also been incrimination against the Bcl-2 family [133]. Transgenic ALS mice showed a prolonged duration of neuronal caspase activation, particularly of caspase 1. As these mice grew older, caspase 1 messenger RNA (mRNA) gradually increased in transcription, followed by caspase 3 mRNA. The transcriptional upregulation of caspase 1 and caspase 3 was delayed in ALS animals treated with the enzymatic caspase inhibitor zVAD-fmk, indicating that these mice undergo a non-cell-autonomous “contagious” apoptotic process [134,135].

### 2.7. Cyclooxygenase-2 (COX-2)

Cyclooxygenase (COX) enzymes catalyze the initial step in prostanoid biosynthesis, playing a crucial role in inflammation and various physiological processes [136,137]. Prostanoids are a broad family of arachidonic acid metabolites, including prostaglandins, prostacyclin, and thromboxanes. Prostanoids are a primary target of non-steroidal anti-inflammatory drugs (NSAIDs). There are two types of COX isoforms: constitutive and inducible. The COX-2 isoform is an inducible isoform rapidly expressed in various cell types in response to growth factors, cytokines, and substances that promote inflammation [28]. Under physiological conditions that are considered normal, COX-2 is displayed in a constitutive manner in many neuronal populations of the mammalian brain. Furthermore, COX-2 mRNA and immunoreactivity were discovered in the granule cells of the dentate gyrus, pyramidal cell neurons in the hippocampus, the piriform cortex, the superficial cell layers of the neocortex, the amygdala, and at low levels in the striatum, thalamus, and hypothalamus of rats [138,139].

However, while discussing the function that COX-2 activity plays in brain diseases, two primary concerns should be considered. The first thing to note is that COX-2 is routinely expressed and plays a role in fundamental brain activities, including synaptic activity, memory consolidation, and functional hyperemia. Second, the response known as “neuroinflammation” is significantly better controlled than the inflammation in peripheral tissues [28]. The location of the two COX isoforms in rat and human tissues has been widely investigated. COX-1 appears to be the sole isoform consistently expressed in most tissues, supporting its role in physiological activities such as stomach cytoprotection and platelet aggregation. It has been demonstrated that COX-1 and COX-2 are expressed in the macula densa cells of the kidneys, testes, and brain when the conditions are normal [140]. COX-1 and COX-2 immunoreactivities are observed in distinct neuronal groupings within the cerebral cortex and hippocampus of the rat brain. The immunoreactivity of COX-1 is shown to be more prominent in some brain regions, including the medulla, pons, and midbrain [138,139]. Similarly, the human brain has mRNAs for COX-1 and COX-2 in various places; however, COX-2 is the predominant isoform, particularly in the hippocampus [141,142]. A significant amount of dispute exists regarding the proportionate contributions that each COX isoform makes to the etiology of Alzheimer’s disease. Researchers have found that the levels of neuronal COX-2 in AD brains are higher, particularly in the early stages [143,144,145], or diminished in the final stage [146]. It is important to note that the upregulation of COX-2 in early Alzheimer’s disease and the reduction of COX-2 in advanced Alzheimer’s disease correlate very well with the levels of prostaglandin E2 (PGE2) in cerebrospinal fluid (CSF). PGE2 levels are higher in individuals who have mild memory impairment (a probable diagnosis of AD) and decrease as the severity of Alzheimer’s dementia increases [147,148]. The expression of COX-2 is observed in neurons, but it is not found in astrocytes or microglia in Alzheimer’s brains [149]. Following status epilepticus, specific groupings of cortical neurons exhibit a rapid rise in the expression of COX-2, a source of inflammatory mediators and a multifunctional neural modulator within the brain. Numerous studies and conjectures have focused on the effects of fast activity-triggered COX-2 activation in neurons [150]. In addition to iNOS, COX-2 has been identified as another significant inflammatory component in the pathophysiology of Parkinson’s disease, as genetic deletion or pharmacological suppression of COX-2 reduces MPTP toxicity to dopaminergic neurons [151].

## 3. Multiple Strategies for Controlling Neurological Disorders by Targeting Associated Enzymes

### 3.1. Naturally Derived Molecules for Controlling Neurological Disorders

Natural compounds derived from plants, marine organisms, and other sources show promising potential for treating neurological disorders like Alzheimer’s, Parkinson’s, and stroke [45,152]. These compounds, including alkaloids, curcumin, resveratrol, and cannabidiol, exhibit neuroprotective properties through various mechanisms such as antioxidant, anti-inflammatory, and anti-amyloid effects [44,153,154]. They can modulate multiple pathways, including Wnt signaling, making them attractive for addressing the complex nature of neurodegenerative diseases [153]. While natural compounds offer potential advantages in safety and efficacy, challenges remain in their development, including limited clinical evidence, quality control issues, and the need for sustainable sourcing [152,155]. Further research, including well-designed clinical trials and interdisciplinary collaboration, is needed to fully harness the therapeutic potential of these natural compounds [42,156].

#### 3.1.1. Plant-Derived Compounds

##### Polyphenols

A growing body of research indicates that polyphenols may offer health benefits. These substances act as potent antioxidants, direct radical scavengers in lipid peroxidation, and interact with several signaling targets involved in biological processes like inflammation and carcinogenesis. They have been linked to pleiotropic biological effects [157]. Synergistic effects and polypharmacology are two well-established landmarks in the world of natural and nature-inspired substances. The entourage impact of various compounds on several biological targets and biochemical pathways frequently builds the foundation for the apparent therapeutic outcome. This is particularly true when considering plant extracts [158].

Examples and Mechanisms (e.g., Resveratrol, Curcumin)

Recent advances in research have provided new impetus and direction for a more detailed analysis of neuro-nutraceuticals, improving our understanding of their essential components and effects on neurological disorders [159]. As numerous studies on resveratrol, curcumin, and other polyphenols demonstrated, assuming a biological effect based on in vitro results is frequently confounding. This point has not yet been covered in this paper, but it is worth emphasizing that in vitro studies using concentrations attainable through oral administration offer essential insights into the mechanisms driving the biological effects of polyphenols and other active compounds. Furthermore, even realistic experimental components might make an in vitro study of the mechanism of action either realistic or implausible. The length of treatment is one example that could be given: upstream binding, phosphorylation, and activation of surface receptors, binding on surface receptors, phosphorylation and activation of transduction factors, and numerous antiradical and antioxidant effects should only be studied for a very brief period, measured in minutes, while the downstream release of cytokines or other cell mediators could be studied after a more extended period of treatment [160].

Efficacy and Safety Profile

Polyphenols, particularly resveratrol, have shown promising cardiovascular benefits in preclinical and clinical studies [161,162,163]. These compounds exhibit antioxidant, anti-inflammatory, and vasodilatory properties, which may potentially reduce cardiovascular disease risk factors [164,165]. However, their low bioavailability and rapid metabolism limit their therapeutic potential [166,167]. Clinical trials have demonstrated moderate improvements in blood pressure and glucose levels in patients with hypertension and diabetes [162,163]. While generally considered safe at specific doses, concerns remain regarding long-term use and potential interactions [164,168]. Further rigorous clinical studies are needed to fully elucidate the therapeutic potential and safety of polyphenols in the prevention and treatment of cardiovascular disease [163,168]. Dietary polyphenols may lower cardiovascular risks and enhance vascular health, preventing arterial stiffness and PVD progression when paired with moderate aerobic exercise. Extensive clinical trials are necessary to maximize their use in the treatment of vascular diseases and validate their therapeutic potential [161].

##### Flavonoids

One of the most prevalent and abundant secondary metabolites, flavonoids are very beneficial to humans for their role in giving plants their hues and the numerous physiologically active components they contain [169]. Research on flavonoid-containing foods has increased significantly because the benefits of flavonoids, such as cancer prevention, have been confirmed [170]. It is well known that flavonoids have a variety of biological actions. The chemicals in this class have been investigated as AChE inhibitors, antioxidants, anti-inflammatory medicines, and, more generally, as prospective scaffolding for creating drugs that counteract neurodegeneration by various pathways [171,172]. A summary of natural molecules and their enzyme targets relevant to neurodegenerative diseases is provided in Table 1.

##### Prominent Compounds (e.g., Quercetin, EGCG)

Flavonoids are polyphenolic compounds found everywhere and make up a large class of natural products [180]. Quercetin’s antidiabetic properties are linked to its ability to inhibit glucose uptake and regulate the mitogen-activated protein kinase pathway [181,182]. Quercetin has been shown to protect against hydrogen peroxide-induced neurodegeneration in pheochromocytoma cells [182]. Another study discovered that quercetin’s mitochondria-targeted effects serve as a method of protection against neurodegenerative disorders [183].

Epigallocatechin gallate (EGCG), a significant physiologically active phenolic component in green tea, has been linked to anticancer activity via many signaling pathways [184]. Some of the mechanisms through which EECG has an anticancer effect include acetylation of amyloid precursor protein (APP) includes promoting apoptosis in human neuroblastoma cells, lowering vascular endothelial growth factor (VEGF) expression in esophageal squamous cells, increasing reactive oxygen species (ROS) with caspase-3 activation, and modifying β-catenin activity to inhibit the proliferation of head and neck cancer cells [185,186,187]. The likely mechanisms for EGCG’s antiosteoporotic action in rats include inhibition of osteoclast cell development, increased mineralization of bone, increased alkaline phosphate action of osteoblast cells, and decreased calcium stone formation brought on by oxidative stress [188,189,190]. EGCG may prevent cells from developing tumors by improving gap junctional communication between cells. According to experimental research, polyphenols may prevent the encouragement of tumor growth through the closure of receptors in the impacted cells [191]. Its inhibitory effects may also eventually hinder angiogenesis and metastasis, the latter stages of carcinogenesis [192].

Impact on Enzyme Activity and Disease Progression

Administration with quercetin has been linked to selective antiproliferative effects and the activation of cell death in cancer cell lines, primarily through an apoptotic mechanism, but not in normal cells [193,194,195]. Quercetin has also been demonstrated to trigger apoptosis by activating caspase-9 and caspase-3 and releasing cytochrome c [196,197,198]. Furthermore, quercetin is believed to have the ability to inhibit PI3K, an enzyme involved in the crucial pathway for cell survival [199,200].

It has been proven that EGCG can inhibit the activity of BACE-1, increase the expression of neprilysin, and repair abnormal synaptic protein levels in the hippocampus and frontal cortex of SAMP8 rats [201]. It was demonstrated that the anti-apoptotic protein Bcl-2 was elevated, and the ratio of Bcl-2 to Bax was increased due to the administration of EGCG. Additionally, a correlation was observed between the decrease in Aβ buildup caused by EGCG and the increased production of neprilysin, the enzyme responsible for the rate-limiting degradation of Aβ [202]. EGCG has been shown to suppress ERK1/2 and Akt-mediated signaling in prostate cancer, block PMA-dependent PKC activation, change the ratio of Bcl-2 family members, and activate caspases [203,204,205].

##### Alkaloids

Alkaloids are beneficial secondary metabolites from plants, fungi, and marine sponges. They have been shown to have therapeutic potential in the treatment of chronic illnesses, such as diabetes, cancer, and neurological diseases. Neurodegenerative diseases are already treated with substances like huperzine A and galanthamine. Because these disorders are complex and caused by oxidative stress, inflammation, and protein aggregation, natural alkaloids with polypharmacological qualities can be used as nutraceuticals and in drug development. Recent developments in naturally occurring alkaloids for treating neurodegenerative diseases are highlighted in this study [45].

Notable Alkaloids (e.g., Berberine)

A quaternary benzylisoquinoline alkaloid with various pharmacological properties, berberine treats heart disease, bacterial infections, inflammation, hypertension, and HIV. Berberine exhibits limited absorption in Phase I clinical trials, but is safe at high dosages [206]. Berberine (BBR) is a plant-derived isoquinoline alkaloid with a wide range of pharmacological activities, including anxiolytic, anti-inflammatory, anti-obesity, anti-fibrotic, anti-diarrheal, anti-hypertensive, anti-arrhythmic, antidepressant, and anti-fibrotic properties. It is applied rather frequently in traditional Chinese medicine [207]. When administered systemically, berberine can move past the blood–brain barrier. The results of preclinical investigations suggest that it may be beneficial in treating a wide range of neuropsychiatric and neurodegenerative neurological disorders. Furthermore, it possesses various pharmacological properties, including anti-hypertensive, antibacterial, anticancer, anti-HIV, and anti-inflammatory activities. Properties that inhibit platelet aggregation, anti-cholinergic activity, anti-protozoal activity, cardiotonic activity, cholagogic activity, antiarrhythmic activity, and choleretic activity are all present [208]. When administered at a daily dose of 1 g for three months, BBR was found to dramatically lower the levels of IL-6 in the serum of patients. BBR can reduce inflammation through several different techniques. There is a connection between the AMPK pathway and the anti-inflammatory and antioxidant properties offered by BBR. Regarding macrophages, inhibiting AMPK can potentially lessen the inhibitory effects of BBR on pro-inflammatory cytokines such as Cox-2 and inducible nitric oxide synthase (iNOS) [209].

Therapeutic Potential and Challenges

BBR’s ability to modulate chemoresistance is due to its various mechanisms, including DNA breaks, drug efflux pumps inhibition, apoptosis and necroptosis modulation, downregulation of multidrug resistance genes, immune response enhancement, and targeting cancer cell pathways like Akt/mTOR, EGFR, MAPK, NF-κB, PARP1, JAK-STAT, and Wnt/β-catenin [210]. Research suggests that BBR can be utilized to treat a range of diseases. Nonetheless, several past studies on the absorption, distribution, metabolism, and excretion of BBR have shown low absorption and rapid metabolism, thereby reducing its bioavailability. In rat studies, BBR exhibits poor oral bioavailability, ranging from approximately 1% (0.68% and 0.36%) [211,212], because of its high distribution in the liver, interaction with P-glycoprotein (Pg-P) efflux pumps, and significant intestinal first-pass elimination. BBR bioavailability issues include low plasma levels, restricted tissue distribution, rapid metabolism, and a short half-life.

#### 3.1.2. Microbial-Derived Natural Molecules

Microbial enzymes have a wide range of therapeutic applications, including the treatment of metabolic disorders, Gaucher’s disease, Parkinson’s disease, and genetic disorders, as well as the removal of toxins, reduction of inflammation, promotion of digestion, and, more recently, the treatment of cancer and infectious diseases. Bacterial enzymes such as L-asparaginase, Serratiopeptidase, Collagenase, Nattokinase, EndoS, α-galactosidase, Prolyl endopeptidase, Cocaine esterase, IdeS, Glutaminase, Vibriolysin, Fibrinolytic enzyme, Methioninase, γ-glutamyl transpeptidase, and Amine oxidase are vital for treatment [213].

#### 3.1.3. Bacterial- and Fungal-Derived Molecules

Due to the limited supply of plant and animal proteases, interest in microbial proteases has grown. Their biochemical diversity and ease of genetic modification make microbes a valuable enzyme source, contributing to over 40% of global enzyme sales [214]. Proteases from microbial sources are preferred over enzymes from plants and animals because they possess nearly all the properties required for biotechnological applications. The genus Bacillus produces the majority of commercial proteases, which are primarily neutral and alkaline. Bacterial neutral proteases operate in a restricted pH range (pH 5 to 8) and have minimal thermotolerance. Because of their intermediate reaction pace, neutral proteases produce less bitterness in hydrolyzed food proteins than animal proteinases, making them ideal for use in food company operations [215]. Fungi, such as *Aspergillus oryzae*, produce alkaline, neutral, and acid proteases with broad substrate specificity and activity across a wide pH range. Solid-state fermentation makes these enzymes efficiently, although they react more slowly and are less heat-tolerant than bacterial proteases. Because acid proteases are pH-specific, they are perfect for creating cheese. In food hydrolysates, neutral proteases help lessen bitterness, whereas alkaline proteases modify food proteins [215].

Moreover, the functional role that viral proteases play in the processing of viral proteins that cause deadly illnesses like cancer and AIDS has made them more significant. Many viruses contain serine, aspartic, and cysteine peptidases [216]. Endopeptidases are the only peptidases encoded by the virus; metallopeptidases are absent. Retroviral aspartyl proteases, necessary for viral assembly and replication, are produced as homodimers inside the polyprotein precursor. Autolysis of the precursor releases the mature protease. Much work is on the expression, purification, and enzymatic investigation of retroviral aspartic protease and mutations. Microbial proteases are recommended to produce potent inhibitors, such as those targeting viral proteases for AIDS treatment, due to their rapid growth, low cultivation requirements, and genetic manipulation for creating enzymes with desirable features.

#### 3.1.4. Marine Organism-Derived Molecules

The global experience of marine pharmacology demonstrates the enormous potential of marine species as raw materials for developing novel pharmacological compounds and medications [217,218]. Moreover, although still largely uncharted, ocean and marine environments are recognized as rich, untapped sources of diverse molecules and macromolecules, including enzymes and bioactive peptides derived from venoms that have the potential to be transformed into extremely valuable biopharmaceutical products. These ecosystems are home to a diverse array of species, including many venomous animals [219]. A marine strain of *Pseudoalteromonas* produces strong α-d-galactosidase with varied applications and research interests [220]. The study demonstrates that Cryptophycins are cytotoxic peptides produced by a lichen cyanobacterial parasite comprising around 25 structurally similar compounds. Arenastatin A (cryptophycin 24), the first marine cryptophycin analog, was isolated from an Okinawan marine sponge after being initially identified in terrestrial bacterial strains [221]. Surprisingly, the discovery of the genes that code for the tailoring enzymes responsible for macrocyclization (Crp TE) and stereospecific epoxidation (CrpE CYP450) was able to tackle two important issues that had been preventing the entire synthesis of those peptolides. Through the use of biocatalysts Crp TE and CrpE, it was found that linearly produced cryptophycins can be efficiently cyclized and epoxidized using a tandem chemoenzymatic methodology [222].

#### 3.1.5. Animal-Derived Natural Compound

The most well-known enzymes of animal origin include pancreatic trypsin, chymotrypsin, pepsin, and rennin [223,224]. Trypsin is a serine protease that hydrolyzes peptide bonds involving lysine and arginine [225]; however, its protein hydrolysates are bitter, making it unsuitable for use in the food industry. It is used to prepare bacterial media and for certain medical applications.

Chymotrypsin is commonly used to accelerate the healing of traumatic, surgical, and orthopedic injuries. Its anti-inflammatory, antioxidant, and anti-infective properties aid in resolving injury-related inflammation and accelerating healing. Chymotrypsin can be employed on its own or in conjunction with other proteolytic enzymes, such as trypsin [226].

Chymotrypsin is a hydrolytic enzyme in pancreatic extracts that breaks down aromatic amino acid linkages. Despite its high cost, it is used in diagnostics and to denature milk protein hydrolysates.

Pepsin is an acidic protease that works in the stomach, similar to HIV-1 protease, to catalyze peptide bond hydrolysis between hydrophobic amino acids. It works best at low pH.

**Figure 2:**
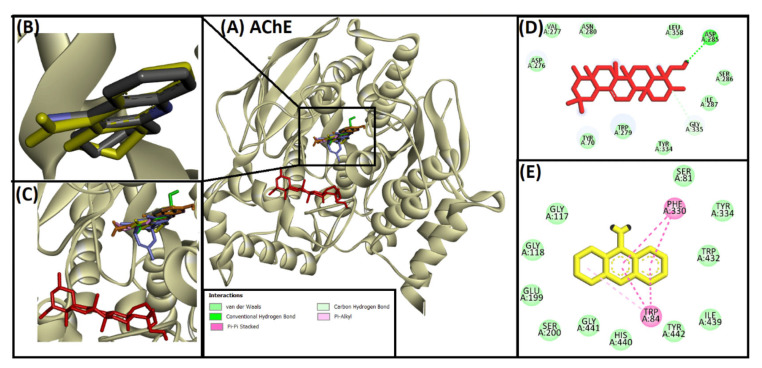
Ligands are docked at the active site of AChE in a superimposed manner (PDB ID: 1ACJ). (**A**) Every docked ligand in the catalytic active site (native ligand: grey; redocked ligand: yellow; alpinetin: pink; calycosin: golden; hydroxytyrosol: green; huperzine A: blue; soyasapogenol B: red). (**B**) The native ligand and redocked native ligand images are superimposed. (**C**) An enlarged picture of every docked ligand. (**D**) Soyasapogenol B’s molecular interactions with amino acid residues are examined. (**E**) Redocked native ligand molecular interaction analysis with amino acid residue. Reprinted from [221], Copyright by author and Licensee MDPI, Basel, Switzerland, distributed under Creative Commons Attribution (CC BY) license (https://creativecommons.org/licenses/by/4.0/).

**Figure 3:**
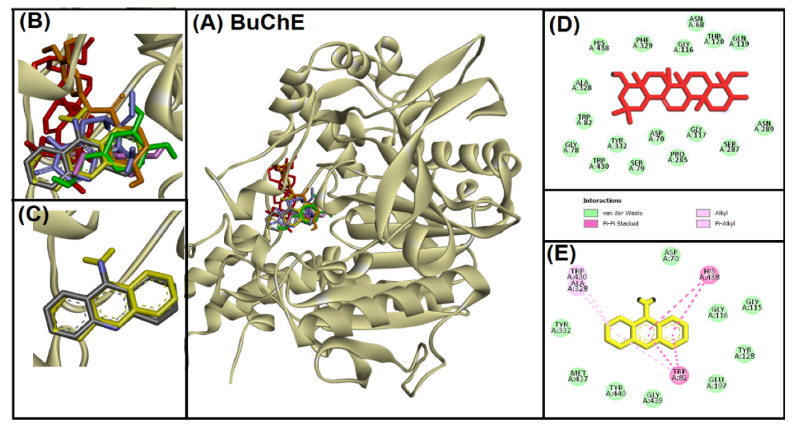
Superimposed image of docked ligands in the active site of BuChE (PDB ID: 4BDS). (**A**) The catalytic active site contains all docked ligands (native ligand: grey; redocked ligand: yellow; Alpinetin: pink; Calycosin: golden; HuperzineA: blue; Hydroxytyrosol: green; Soyasapogenol B: red). (**B**) A zoomed-in image of all docked ligands. (**C**) A zoomed-in image of the native ligand is superimposed over the redocked native ligand. (**D**) Molecular interaction study of Soyasapogenol B with amino acid residues. (**E**) Molecular interaction study of a redocked native ligand with amino acid residues. Reprinted from [227]. Copyright © 2022 by the authors and Licensee MDPI, Basel, Switzerland, distributed under the Creative Commons Attribution (CC BY) license (https://creativecommons.org/licenses/by/4.0/).

Rennin (Chymosin) is a dairy enzyme that transforms κ-casein into curd-forming components. Its unique cleavage activity is vital in cheese manufacturing [215]. Another study proposed that Soyasapogenol B showed promise as a natural inhibitor of AChE (Figure 2) and BuChE (Figure 3, with favorable physicochemical qualities, binding stability, and safety profile, indicating its potential for treating neurodegenerative illnesses such as Alzheimer’s disease [227].

### 3.2. Chemically Synthesized Molecules for Controlling Neurological Disorders by Targeting Associated Enzymes

Although several synthetic formulations are used to treat AD, nanoformulations appear to boost their effectiveness. By encouraging neuronal survival and regulating neurogenesis, erythropoietin (EPO), a hematological factor, may be a neuroprotective agent in AD. However, due to its considerable molecular weight, hydrophilicity, and rapid blood clearance, EPO transport to the central nervous system is complex and occurs in insufficient quantities [228]. Research conducted in vivo has indicated that EPO-SLN may protect the brains of animals from brain injury induced by intra-hippocampal injection. This was demonstrated by the remarkable recovery of spatial recognition memory in rats treated with free EPO. Nicotinamide, a histone deacetylase blocker, is another synthetic medicine with poor absorption rates. Additionally, it has been demonstrated in preclinical trials to be beneficial in slowing AD [228]. Rivastigmine SLN, although lipidic, was shown to have a higher rate of drug diffusion compared to a drug solution that included the drug in crystalline form, according to the findings of another investigation. Since rivastigmine SLN does not affect cell necrosis or nasociliary disruption, it can be administered nasally [229]. For effective transport across the blood–brain barrier (BBB), n-butyl cyanoacrylate nanoparticles encapsulating clioquinol, a quinoline derivative known to solubilize amyloid plaques in extracellular synaptic regions during the early stages of Alzheimer’s disease, may be employed [230]. It is possible that administering essential fibroblast growth factor (bFGF) into the hippocampus, another synthetic medication, could prevent neuronal degeneration and improve learning difficulties in rats with Alzheimer’s. Intranasal administration of Solanum tuberosum lectin-coupled polyethylene glycol (PEG-PLGA) nanoparticles has the potential to effectively boost the direct transport of bFGF into the rat brain, while simultaneously reducing the number of peripheral adverse effects [231].

Examples of synthetic enzyme inhibitors with potential therapeutic effects are summarized in Table 2.

#### 3.2.1. Small-Molecule Inhibitors

**Figure 4:**
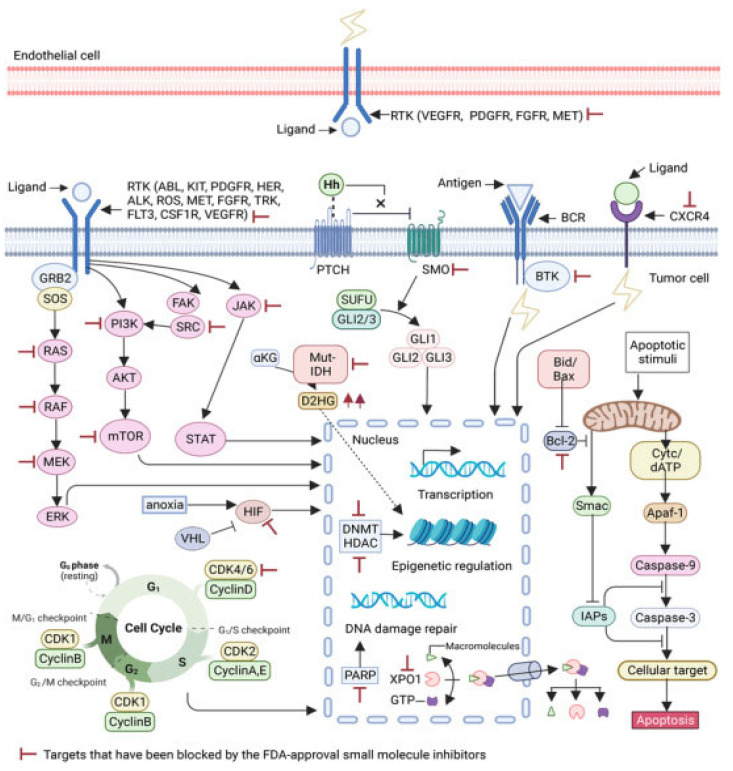
The Food and Drug Administration of the United States has approved small-molecule inhibitors that target cancer and the processes that cause it. Small-molecule inhibitors that have been approved by the Food and Drug Administration in the United States target a wide range of functions, such as nuclear transport, blood vessels, apoptosis, DNA transcription, DNA damage repair, cell surface receptors, signaling pathways, and epigenetic modifications. Reprinted from [238], Copyright, © 2022 by the Authors. MedComm is published by Sichuan International Medical Exchange & Promotion Association (SCIMEA) and John Wiley & Sons Australia, Ltd., under the terms of the http://creativecommons.org/licenses/by/4.0/.

Small-molecule inhibitors have a promising future because they can explore novel and challenging drug targets, identify promising candidates for clinical evaluation using predictive non-clinical models, and deliver precision medicine through dynamic clinical trial interventions with liquid biopsies [237]. Compared to traditional drugs, targeted therapy offers advantages in terms of selectivity, efficacy, and tolerance from the patient’s perspective. Small-molecule inhibitors are among the targeted cancer medications utilized most frequently (Figure 4). Small-molecule inhibitors have been the subject of numerous efforts to be manufactured because of the numerous benefits they offer, including the ability to target a wide variety of targets, the simplicity of administration, and the ability to reach the central nervous system [238]. The protein kinase inhibitors comprise most small-molecule inhibitors [239,240,241]. Small-molecule inhibitors can selectively disrupt cell signaling by their ability to attach to a particular target. A few dysfunctions associated with growth, survival, apoptosis, differentiation, cancer metabolism, and even immune control are responsible for a significant portion of cancers [242,243]. Selective small-molecule and small-molecule nonkinase inhibitors are the two subclasses of selective small-molecule inhibitors [237]. Protein kinase inhibitors are the most common type of small-molecule inhibitors. The number of kinases that have IC_50_ values of inhibitory activity that are lower than 10 nM is the criterion that determines whether a protein kinase inhibitor is categorized as a multikinase inhibitor or a selective small-molecule kinase inhibitor [244].

The research currently being conducted on ABL1 kinase inhibitors primarily focuses on three key areas of interest. First, there is still a significant interest in novel ABL1 kinase inhibitors. Both flumatinib and radotinib are examples of ABL1 kinase inhibitors of the second generation. Both of these drugs have been approved for use in China and Korea for the treatment of Ph-positive CML-CP. It has also been demonstrated that both of these drugs are more effective than imatinib when used as the initial treatment for patients who have just been diagnosed with CML-CP [245,246]. There has been a significant improvement in the prognosis for many malignancies due to the development and approval of small-molecule inhibitors, which have significantly altered clinical therapy for many tumors [247]. KIT kinase inhibitors are still the subject of significant study. Dasatinib, nilotinib, and ponatinib are all examples of ABL1 kinase inhibitors that inhibit c-KIT in a manner comparable to that of imatinib. Ponatinib demonstrated efficacy in advanced GISTs with previous TKI failure, particularly in the subtype comprised of KIT exon 11 mutations, according to the findings of a phase II trial with a single arm [248]. A phase III clinical trial demonstrated that nilotinib was not superior to imatinib in the treatment of first-line GISTs [249]. Imatinib was the first drug tested in clinical trials to treat melanoma. Nilotinib, dasatinib, and regorafenib were the subsequent drugs of choice. With an overall response rate (ORR) ranging from 16 to 29%, it was discovered that imatinib effectively treated melanoma patients who had KIT amplification and/or mutations in phase II clinical studies [250,251,252]. Melanoma patients with c-KIT mutations are being recruited for a phase II research study to determine whether regorafenib is effective as a second-line treatment (NCT02501551). A recent development for melanoma with c-KIT gene alterations is the combination of KIT inhibitors and PD-1 inhibitors (NCT05274438). Additionally, ripretinib is evaluated in individuals with advanced cancers through a phase I trial (NCT02571036). First and foremost, the research and development of new KIT kinase inhibitors is still ongoing. Masitinib is a form of TKI that is highly effective and highly selective. It can inhibit both the juxtamembrane mutant and the wild-type c-Kit receptor. Masitinib was evaluated as the first-line treatment for advanced GIST in a phase II study (NCT00998751), which found that it was comparable to imatinib regarding response and safety [253]. Masitinib was evaluated as the first-line treatment. Not only does the pan-KIT mutant kinase inhibitor AZD3229 target PDGFRα, but it also targets itself. Compared to imatinib, it is 15–60 times more potent in inhibiting KIT primary mutations, demonstrating low nanomolar potency against a wide variety of secondary mutations. However, the preclinical stage is still being carried out [254]. Expanding the indications of already available drugs and creating new KIT inhibitors are two of the primary research approaches being pursued [238].

In treating human breast, ovarian, and colon cancers, the small-molecule CDK4/6 inhibitor SHR6390 is administered orally and is characterized by its effectiveness and selectivity. According to the findings of the clinical investigation, the moderate CYP3A4 inducer efavirenz significantly impacts the pharmacokinetic behavior of SHR6390. The exposure level AUC of SHR6390 demonstrates a moderate level of induction effect. CYP3A4 moderate inducers should not be used in conjunction with one another in clinical therapy, as this is not recommended. Oral administration of 150 milligrams of SHR6390, in conjunction with 600 milligrams of efavirenz, was found to be well tolerated in healthy volunteers [255].

An oral small-molecule JAK inhibitor called tofacitinib is approved to treat moderate to severe ulcerative colitis (UC). Its effectiveness and security have been shown in phase III clinical trials backed by empirical evidence. This study reports a severe aggravation of an 18-year-old female patient who had a 1-year history of left-sided ulcerative colitis that was unresponsive to vedolizumab and infliximab. After starting tofacitinib, the patient experienced acute kidney damage (grade 1 KDIGO) and persistent eosinophilia (3000 cells/mm^2^) after 6 days, which led to a brief suspension and a quick return to typical lab abnormalities. Eosinophilia (2000 cells/mm^3^) returned after rechallenge, leading to a permanent cessation. Three months after starting tofacitinib suspension, there was no relapse of eosinophilia, and clinical and biochemical remission was attained with high-dose corticotherapy, ustekinumab (every four weeks), and tacrolimus [256].

In a case study, a 30-year-old male with an NBN germline mutation and ROS1-positive lung cancer showed improvement while receiving platinum-based chemotherapy and ROS1 inhibitors. Stable disease (SD) was achieved with a progression-free survival (PFS) of more than 12 months and an overall survival (OS) of 23 months thus far after treatment was changed to sintilimab with niraparib. With manageable side effects, the combination showed effectiveness in treating resistant cancers with HRR gene alterations, offering a possible approach for individualized treatment in cases like this. To validate this strategy, more clinical trials are required [257].

#### 3.2.2. Peptide Inhibitors

Peptide therapeutics show promise for treating neurological disorders due to their high specificity and low toxicity [258,259]. However, their application faces challenges, primarily rapid degradation and poor blood–brain barrier (BBB) penetration [260,261]. Strategies to overcome these limitations include the use of D-amino acids, chemical conjugation, and encapsulation to extend peptide half-lives [258]. BBB penetration can be improved through receptor-targeted peptides, cell-penetrating peptides, and nanoparticle-based delivery systems [261,262]. Peptides targeting metal ion dyshomeostasis and protein aggregation have shown potential in the treatment of neurodegenerative diseases [263,264]. Despite these advancements, challenges remain in achieving therapeutically effective concentrations in the brain and addressing potential side effects [261,265].

### 3.3. Advanced Drug Delivery Systems

Advanced drug delivery systems are designed to (1) regulate the rate at which drugs are released from formulations or dosage forms, (2) regulate the rate at which drugs are delivered to absorbing membranes and surfaces, and (3) regulate the rate at which drugs are released at specified sites. The two most crucial characteristics of the different drug delivery methods are the control of drug release and the power of body distribution [266]. Several chemopreventives have demonstrated limited efficacy in preclinical and clinical studies due to their low bioavailability, resulting in subtherapeutic concentrations at the target site. To address bioavailability concerns, new drug delivery systems that offer localized or targeted distribution of these medicines may be a more realistic treatment choice. Various medication delivery techniques, including nanoparticles [267,268], microparticles [268,269], liposomes [270,271], and implants [272], have been found to significantly improve the preventive/therapeutic efficacy of numerous chemopreventives by enhancing their bioavailability and targeting abilities.

Efforts to enhance oral drug bioavailability have advanced alongside the growth of the pharmaceutical industry. With the rise in the number of drugs and chemical diversity, innovative strategies have become essential for developing effective oral therapeutics. The main parameters affecting oral bioavailability are water solubility, drug permeability, rate of dissolution, presystemic metabolism, sensitivity to efflux mechanisms, and first-pass metabolism. Among these characteristics, poor permeability and poor solubility are the most frequent causes of low oral bioavailability [273,274,275]. A comprehensive awareness of the factors underlying low bioavailability and preferred absorption pathways, and how the various techniques affect drug metabolism and the desired pharmacokinetic profile, is essential to choosing the best approach for a given drug or therapeutic candidate [276].

Prodrugs are inactive drug derivatives that undergo enzymatic or chemical hydrolysis inside the body to transform into active drugs. To increase bioavailability, prodrugs are usually created by covalently attaching a chemical moiety to a drug that modifies the drug’s physicochemical properties. This covalent bond should be moderately labile and developed to be cleaved, releasing the active drug once the prodrug is absorbed into the systemic circulation; this method has proven helpful in many types of drugs [277].

The drug’s solubility is one of the most important aspects determining pharmaceutical compounds’ bioavailability and therapeutic effectiveness. A lack of solubility, particularly in the gastrointestinal tract, can hinder efficient absorption. In pharmaceutical substances, magnetic nanoparticles (MNPs) have emerged as a promising technique for overcoming these obstacles, as they can enhance the stability, solubility, and therapeutic effectiveness of drugs [278]. MNPs possess unique properties that enhance drug solubility in the gastrointestinal tract (GI). Because of their unique qualities, MNPs can reduce the amount of medication soluble in the gastrointestinal tract (GI). They work better with poorly soluble drugs because of their small size, large surface area, and coating-functionalization capabilities. For instance, Curcumin-loaded PLA-HA/Fe3O4 increased the drug’s solubility in the digestive system, which could potentially cure colorectal cancer by increasing the bioavailability of curcumin, a substance that is renowned for its low solubility [279].

In addition, the ability of MNPs to cross the blood–brain barrier, their capacity for targeted medicine delivery, and their application in various neuromodulation methods have contributed to their growing significance in treating neurological illnesses. The use of magnetic micro-hydrogels as a treatment for neurological diseases in the central nervous system is a potential option. These microparticles, guided by external magnetic fields, provide precise targeting, enabling more effective neuromodulation. Compared to traditional SPIONs, these micro-hydrogels show superior neuron activation capabilities, offering new treatment options for neuropsychiatric disorders [280].

In addition to their use in Parkinson’s disease, magnetic nanoparticles (MNPs) have been investigated for treating neurological conditions such as Alzheimer’s. With superparamagnetic iron oxide nanoparticles (SPIONs), extracellular vesicles from human forebrain organoids have been labeled, allowing for real-time MRI tracking to track the course of the disease and evaluate the efficacy of treatment [281].

Furthermore, drug solubility in intestinal fluid and permeability across the intestinal membrane can act as rate-limiting processes in drug absorption, resulting in limited bioavailability [282]. These disadvantages are commonly found in administering peptide or protein-based drugs [283]. Therefore, intravenous (IV) injection is considered one of the most effective methods for delivering protein-based drugs, achieving up to 100% bioavailability, precise dosage, and avoiding hepatic metabolism [284]. However, IV administration has drawbacks like invasiveness, patient discomfort, low compliance, and high expenditures associated with sharps waste disposal [285,286].

The subsequent approach for improving transdermal delivery of drugs is the use of vesicles and their counterparts. In 2008, this process was categorized as a second-generation transdermal product [287]. The main constituents of nanovesicles, spherical, nanoscale bilayer structures, are lipids or structurally similar amphiphilic substances like surfactants [288]. Some nanovesicles, such as liposomes, ethosomes, transfersomes, niosomes, and phytosomes, enable transdermal administration. The primary ingredient utilized in the formulation determines the classification of these nanovesicles [289]. Microneedle technology platforms have demonstrated greater versatility than previous transdermal systems, with the potential for both therapeutic drug monitoring and intradermal delivery of drugs and biotherapeutics. According to this, microneedles have emerged as a promising tactic for enhancing transdermal administration methods [289].

The other study focuses on generating hybrid compounds of caffeic acid and resveratrol and assessing their capacity to inhibit two essential enzymes involved in Alzheimer’s disease: BACE 1 and acetylcholinesterase. Molecular docking studies were carried out to investigate the interactions between these synthetic chemicals and the enzymes’ active regions. Both in vitro testing and molecular docking studies showed that the compounds were effective, particularly in suppressing BACE 1 and AChE. These findings indicate that these hybrid compounds hold substantial promise as possible anti-Alzheimer’s medicines, with their design inspired by molecular docking interactions [290].

#### 3.3.1. Nanomaterials-Mediated Control of Neurological Disorders by Associated Targeting Enzymes

Nanoparticles offer significant advantages over conventional drug delivery systems, addressing key challenges in therapeutic efficacy. They enhance drug solubility, bioavailability, and targeted delivery while reducing side effects [291,292]. Nanocarriers can overcome biological barriers, including nonspecific distribution and inadequate accumulation at disease sites [293]. Various types of nanoparticles, such as polymeric, metallic, and liposomal, have been developed for targeted drug delivery in cancer, infectious diseases, and neurological disorders [294,295]. Surface modification with targeting ligands improves specificity and cellular uptake [296]. Nanoparticles also show promise in overcoming drug resistance mechanisms in cancer therapy [297]. Biointerface engineering of nanocarriers can further optimize drug delivery and promote personalized medicine approaches [298]. These advancements in nanomedicine present new opportunities for enhancing treatment outcomes across various diseases.

Over the past few decades, nanoparticles have demonstrated a strong ability to reduce inflammation. These anti-inflammatory benefits result from the elevated surface area-to-volume ratio, leading to nanoparticles being more likely to block cytokines, enzymes, and other components related to inflammatory processes. Zinc oxide, selenium, iron oxide, gold, CeO, silver, magnesium oxide, and copper are among the various nanoparticles that have been discovered to possess anti-inflammatory properties both in vitro and in vivo (Table 3) [299].

Having been considered, synthetic drug nanoformulations such as nicotinamide, erythropoietin, donepezil, galantamine, memantine, clioquinol, rivastigmine, estradiol, bFGF, and tarenflurbil have demonstrated a variety of effects on AD by promoting neurogenesis, enhancing bioavailability, decreasing protein aggregation, and reducing dysregulated inflammation, apoptosis, and oxidative stress. The preclinical data support the use of synthetic medication nanoformulations to treat AD [308]. Nanoparticles’ inherent antioxidant qualities are negligible, and the majority of observed antioxidant benefits have been linked to the antioxidant chemicals they contain. By increasing antioxidant chemicals’ bioavailability, solubility, and BBB permeability, nanoparticles have improved their effectiveness and performance [309]. On the other hand, certain oxide nanoparticles scavenge the ROS and RNS and activate antioxidant mediators to produce intrinsic antioxidant activity. The effects of many enzymes that help reduce oxidative stress, like phosphatase, catalase, and superoxide dismutase (SOD), can be accurately mimicked by cerium oxide (CeO) nanoparticles [310]. Gil et al. examined the advantageous and antioxidant properties of nanocrystalline cerium dioxide (CeO2) under the condition that it is conjugated with CAT and SOD enzymes. The findings showed that nanoceria and antioxidant enzymes were sufficiently stable [311]. This discovery was made possible by the synergistic increase in antioxidant activity. Studies have shown that zinc oxide nanoparticles have anti-inflammatory activity by decreasing the pro-inflammatory cytokines TNF-α and IL-1β. These cytokines downregulate inflammatory responses and reduce mast cell proliferation and differentiation. Furthermore, zinc oxide nanoparticles were found to reduce the quantity of caspase-1 in activated mast cells, inhibit NF-κB signaling, and lipopolysaccharide-induced NF-κB, and significantly reduce the cytosolic breakdown of IκBα, as well as the production of malondialdehyde, IL-1β, and TNF-α [299,312,313]. According to the findings of these applications, nanoparticles have been employed in numerous studies to enhance the neuroprotective properties of various medications, both in vitro and in vivo.

#### 3.3.2. Nanomaterials and Action Mechanisms Targeting Neurodegenerative Enzymes

**Figure 5:**
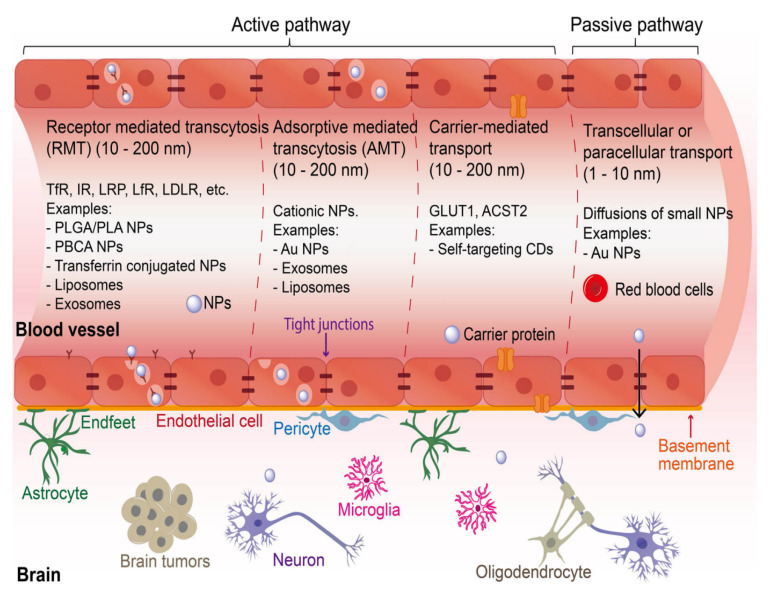
As shown schematically in the figure, nanoparticles (NPs) penetrate the BBB through active and passive transport processes. The three active mechanisms are carrier-mediated transport, adsorptive-mediated transcytosis, and receptor-mediated transcytosis (RMT). Diffusion occurs both transcellularly and paracellularly in passive channels. Key BBB elements, including endothelial cells, tight junctions, pericytes, astrocytes, and other brain cells, are highlighted in the picture, along with a list of nanoparticle kinds (such as PLGA/PLA NPs, liposomes, and exosomes) that are pertinent to each transport pathway. Reprinted from [319], under the https://creativecommons.org/licenses/by/4.0/.

The blood–brain barrier (BBB) is an essential capillary endothelial cell contact that controls the flow of chemicals between the margins of the brain and the brain itself (Figure 5), protecting against infectious agents. Many components comprise the blood–brain barrier (BBB), such as close interactions between extracellular matrix proteins, endothelial cells, pericytes, astrocyte endfeet, microglia, interneurons, and basement membranes. Together, these components perform their functions to maintain the selective permeability of the BBB (Figure 5). The integrity of the barrier is dependent on tightly controlled junctions, permeability controlled, and the specific expression of endothelial cell receptors and transporters [314,315,316]. Through adsorptive and receptor-mediated transcytosis, which is enabled by specific receptors and transporters, molecules can traverse the blood–brain barrier. Astrocytes are a classification of glial cells that interact with neurons and capillaries in the brain. Several extracellular matrix (ECM) proteins (e.g., heparan sulfate proteoglycans, laminin, and type IV collagen) are located in the basement membrane, which is the membrane that surrounds endothelial cells and pericytes [317,318].

## 4. Conclusions and Future Perspectives

The complex disorder and limited treatment choices of neurological disorders, especially neurodegenerative diseases like Parkinson’s and Alzheimer’s, pose a serious global problem. By tackling important pathways like oxidative stress, neuroinflammation, and protein aggregation, enzyme-targeted medicines have great potential to slow disease progression. Synthetic medications, such as enzyme inhibitors and tiny molecules, offer precision and efficacy, whereas natural compounds, such as curcumin, resveratrol, and quercetin, offer safe and multi-target potential.

Future therapeutic approaches for neurodegenerative diseases should concentrate on creating multi-target strategies that combine synthetic and natural molecules with cutting-edge drug delivery technologies, like nanoparticles and microneedle technologies, to improve efficacy and get around bioavailability issues. Customized treatments, real-time monitoring, and drug design optimization to target important enzymes like sphingomyelinases, AChE, and *BACE1* are all made possible by precision medicine, which is fueled by nanotechnology and artificial intelligence. While creative formulations and non-invasive delivery techniques guarantee effective transport across the blood–brain barrier, investigating neglected resources like marine-derived chemicals and microbial enzymes may provide novel therapeutic agents. Addressing the intricate pathologies of neurological illnesses and enhancing patient outcomes will require translational research and well-defined regulatory frameworks to bridge preclinical developments to clinical applications. One promising strategy for treating neurodegenerative illnesses is the combination of natural and synthetic chemicals, nanotechnology, and sophisticated delivery systems. Although there has been a lot of progress, future work should concentrate on multi-target treatments, enhancing bioavailability, and utilizing precision medicine technologies to get past current obstacles. The solution to reducing the worldwide burden of neurodegenerative diseases lies in ongoing research and innovation. It is also important to recognize that promising preclinical results do not always translate into clinical success due to species differences, pharmacokinetic variability, and unexpected safety issues.

## Figures and Tables

**Table 1:** Natural molecules targeting enzymes associated with the progression of neurodegenerative diseases.

Name of Molecules	Sources	Chemical Structure	Active Concentration	Target Enzymes	Name of ND	Mechanism of Action	In Vivo Test	References
Curcumin	Turmeric (Curcuma longa)	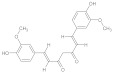	0 mg/kg body weight (i.p.) or 2.0 g/kg diet	Caspase-3 and caspase-9	Cerebral ischemia/reperfusion injury	Reduces oxidative stress, decreases lipid peroxidation, restores mitochondrial function, and inhibits apoptosis by reducing caspase-3 activation	Tested in Mongolian gerbils (global cerebral ischemia model)	[173]
Resveratrol	Grapes, red wine, and certain berries	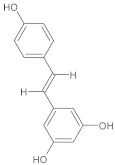	30 mg/kg/day	Calcium-dependent enzymes, xanthine oxidase, nitric oxide synthase (NOS), phospholipase A2 (PLA2)	Kainic acid-induced neurotoxicity (excitotoxicity in the hippocampus)	Acts as a free radical scavenger; reduces oxidative stress, neuronal death, and glial activation caused by kainic acid	Tested in adult Sprague-Dawley male rats treated with kainic acid (8 mg/kg) for 5 days	[174]
Galanthamine (GAL)	Amaryllidaceae plants	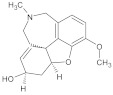	3 mg/kg (1/10 of LD50)	Acetylcholinesterase (AChE)	Alzheimer’s disease (AD)	AChE inhibition, antioxidant activity	Male and female ICR mice (6 weeks old, 25–35 g, behavioral analysis, hematological and biochemical tests	[175]
4b (Hybrid)	GAL and CU hybrid	[Structure of 4b]	5 mg/kg (1/10 of LD50)	Acetylcholinesterase (AChE)	Alzheimer’s disease (AD)	AChE inhibition, antioxidant, BBB permeable	Male and female ICR mice (6 weeks old, 25–35 g, acute toxicity, short-term toxicity, behavioral studies	[175]
Apigenin	Found in plants such as parsley, celery, and chamomile	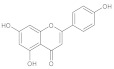	20 mg/kg (oral administration)	iNOS, COX-2, MAPK (p38, JNK)	Neurodegenerative diseases (Alzheimer’s disease, Parkinson’s disease)	Anti-inflammatory effect, inhibition of iNOS expression, reduction of NO production, modulation of microglial activation, inhibition of MAPK signaling pathways (p38, JNK)	MCAO mice model, TTC staining, OX-42 immunohistochemistry	[176]
α-Bisabolol	Found in the essential oil of *Matricaria chamomilla* (chamomile)	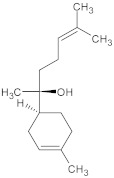	100 mg/kg (low dose), 200 mg/kg (high dose)	Acetylcholinesterase (AChE), α-Amylase	Diabetic Alzheimer’s disease (Type 2 Diabetes with cognitive decline)	Neuroprotective, improves spatial memory, reduces blood glucose, inhibits AChE, antioxidant activity, and anti-inflammatory	Morris water maze, open field test, acetylcholinesterase activity, and enzymatic antioxidants in rat brain	[177]
Lycopene	Found in tomatoes, watermelon, and red fruits	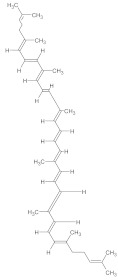	Varies (commonly 5–10 mg/kg)	NF-κB, caspase-3, inflammatory cytokines (TNF-α, IL-1β, TGF-β)	Alzheimer’s disease (AD), neuroinflammation	Reduces pro-inflammatory cytokines (TNF-α, IL-1β, TGF-β), inhibits NF-κB and caspase-3, prevents Aβ-induced mitochondrial dysfunction, improves learning and memory functions	ICV Aβ1–42-induced rats (Morris water maze, elevated plus maze)	[178]
Ruscogenin	Derived from *Ophiopogon japonicus* (a traditional Chinese medicinal plant)	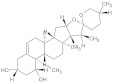	10–20 mg/kg	Inflammatory cytokines, oxidative stress markers	Parkinson’s disease (PD)	Reduces oxidative stress and inflammation, improves motor function, protects dopaminergic neurons in the substantia nigra	MPTP-induced PD mice (footprint test, grip strength, rota rod, histopathology)	[179]

**Table 2:** Synthetic molecules targeting enzymes associated with the progression of neurodegenerative diseases.

Name of Molecules	Chemical Structure	Active Concentration	Target Enzymes	Name of ND	Mechanism of Action	In Vivo Test	References
Diazepam	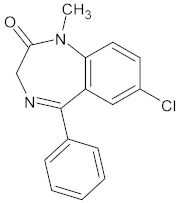	Dose range: 0.1–1 mg/kg.	Targets GABA-A receptors (enhances inhibitory neurotransmission).	Alzheimer’s disease (AD).	Enhances GABAergic neurotransmission, reducing anxiety but impairing cognitive processes like memory due to over-inhibition.	Animal Model: C57Bl/6 female mice.	[232]
Scopolamine	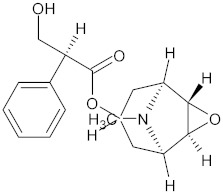	Dose range: 0.1–1 mg/kg.	Targets muscarinic acetylcholine receptors (blocks cholinergic signaling).	Schizophrenia and autism.	Antagonizes muscarinic acetylcholine receptors, disrupting cholinergic signaling critical for memory formation.	Animal Model: C57Bl/6 female mice.	[232]
Donepezil	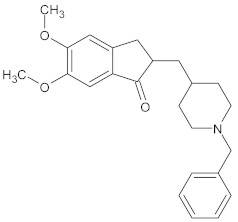	Dose: 1 mg/kg.	Targets acetylcholinesterase (inhibits breakdown of acetylcholine, enhancing cholinergic signaling).	Altered sociability is linked to neurotransmitter imbalances.	Inhibits acetylcholinesterase, increasing synaptic acetylcholine levels, thereby reversing memory deficits caused by cholinergic antagonism.	Animal Model: C57Bl/6 female mice.	[232]
Haloperidol	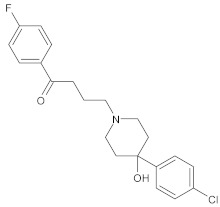	Doses: 0.06, 0.12, 0.24 mg/kg (chronic treatment for 102 days).	Target: Dopamine D2 receptors (antagonist).	Parkinson’s disease (PD): Parkinsonian-like motor impairments (e.g., bradykinesia).	Chronic D2 receptor blockade reduces dopamine signaling in the basal ganglia, leading to motor impairments characteristic of Parkinsonian effects and tardive dyskinesia.	Rats. Duration: 102 days (chronic haloperidol treatment), with drug testing conducted every second or third day.	[233]
Trihexyphenidyl	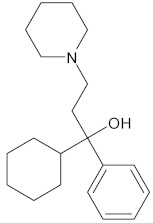	Doses: 0.15–1.0 mg/kg.	Target: Muscarinic acetylcholine receptors (antagonist).	Effect: Reduced some of haloperidol’s effects on licking behavior, Parkinson’s disease.	Mechanism: Modulate serotonin receptor activity to influence motor and behavioral outcomes linked to haloperidol-induced effects.	Rats. Duration: 102 days (chronic haloperidol treatment), with drug testing conducted every second or third day.	[233]
Trihexyphenidyl	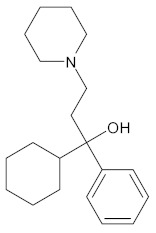	1 mM (local via microdialysis), 1.5 mg/kg (systemic, i.p.). L-dopa: 2 μM for 20 min (local application).	Trihexyphenidyl: Muscarinic acetylcholine receptors (non-selective antagonist). - L-dopa: Aromatic L-amino acid decarboxylase (AADC) for dopamine synthesis.	Parkinson’s disease (PD).	Trihexyphenidyl: Blocks muscarinic acetylcholine receptors, reducing cholinergic overactivity. Attenuates L-dopa-induced dopamine release systemically in intact striatum. - L-dopa: Precursor to dopamine, converted by AADC in dopaminergic neurons.	Hemi-Parkinson rat model with unilateral 6-hydroxydopamine lesion of nigrostriatal pathway.	[234]
(R,S)-trihexyphenidyl		1–30 μM.	THP more effectively counters cholinergic crisis, seizures, and neuropathology triggered by OP-induced AChE inhibition. - THP blocks mAChRs and NMDARs in the brain, and inhibits α7 nAChRs.	Parkinson’s disease (PD).	- Suppresses glutamatergic synaptic transmission via an action potential-dependent mechanism. - Independent of NMDAR, mAChR, and α7 nAChR inhibition.	The study used primary hippocampal cultures derived from rats to investigate the effects of (R,S)-trihexyphenidyl (THP) on synaptic transmission.	[235]
LY2886721	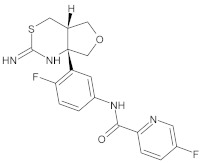	EC50: ~10 nM in PDAPP neuronal cultures, 18.5–19.7 nM in HEK293Swe cells.	*BACE1*	Alzheimer’s disease (AD).	Inhibition of *BACE1* to reduce Aβ production and lower amyloid levels.	PDAPP transgenic mice (APPV717F mutation).	[236]

**Table 3:** Nanomaterials targeting enzymes associated with the progression of neurodegenerative diseases.

Nanomaterials	Natural Products Used in the Synthesis of NPs	Active Concentration	Target Enzymes	Name of ND	Mechanism of Action	In Vivo Test	References
Selenium NPs (SeNPs)	*Aloe vera*, *Prunus amygdalus*, *Vitis vinifera*, *Allium sativum*, *Dillenia indica*, *Roselle* plant, *Cinnamomum zeylanicum*, fresh citrus, lemons	5–10 μg (non-toxic), 20–25 μg (toxic effects)	Superoxide dismutase (SOD), Glutathione peroxidase (GSH-PX	Parkinson’s disease (PD), Alzheimer’s disease (AD)	Antioxidant activity, ROS reduction, inhibition of tau hyperphosphorylation, interaction with Aβ aggregates	MPTP-induced PD model	[300]
PEG-AuNPs (polyethylene glycol–gold nanoparticles)	Anthocyanins	12 μg/g/day for 14 days	p-PI3K, p-Akt, p-GSK3β	Alzheimer’s disease	Prevented tau hyperphosphorylation, protected synaptic proteins, inhibited apoptosis, and reduced neurodegeneration	Aβ1–42 mouse model of AD	[301]
HS-AuNPs (Hibiscus sabdariffa–synthesized gold nanoparticles)	*Hibiscus sabdariffa* extract	5 mg/kg and 10 mg/kg b.w. for 14 days	Acetylcholinesterase, monoamine oxidase, adenosine deaminase, COX-2, BACE-1	Alzheimer’s disease	Reduced oxidative stress, improved antioxidant enzyme activities (SOD, GPx, GSH), decreased inflammatory markers (COX-2, BACE-1), and ameliorated memory and learning deficit	AlCl3-induced AD model in Wistar rats	[302]
CUR-LF NPs (Curcumin–Lactoferrin Nanoparticles)	Curcumin (CUR), Lactoferrin (LF)	10 mg/kg	Aβ25–35-induced oxidative stress	Alzheimer’s disease	Antioxidant, anti-inflammatory, and neuroprotective effects. CUR-LF NPs protect neurons from oxidative damage and apoptosis and improve bioavailability	Rats were administered CUR-LF NPs via the IN and IV routes. Pharmacokinetic studies were performed to evaluate brain accumulation	[303]
P-80-LYC-PSCNP (Polysorbate-80– Lycopene– Phosphatidylserine–Chitosan Nanoparticles)	Lycopene (LYC), Polysorbate-80 (P-80), Phosphatidylserine, Chitosan	5 mg/kg LYC-equivalent dose	Catalase (CAT), Superoxide dismutase (SOD), Glutathione peroxidase (GPx)	Neurodegenerative diseases (oxidative stress-related	Antioxidant properties through improved enzymatic activity, alleviates oxidative stress by delivering Lycopene across the blood–brain barrier (BBB), reducing cognitive and behavioral impairments	Streptozotocin-induced oxidative stress model (SOSM), behavioral despair test, biochemical assays for enzymatic activity	[304]
AuNPs-piperine	Piperine	10 μM	Likely oxidative stress-related enzymes (e.g., SOD, CAT)	Parkinson’s disease (PD)	Neuroprotection against PQ-induced oxidative stress	in Drosophila	[305]
PM-AuNPs	*Paeonia moutan* root extract	20 mg/kg (in vivo)	iNOS, COX-2, Tyrosine hydroxylase (TH)	Parkinson’s disease (PD)	Reduces neuroinflammation and improves motor coordination	In Parkinson-induced C57BL/6 mice	[306]
Gold Nanoparticles (AuNPs)	*Cinnamomum verum* (Cinnamon) extract	5 mg/kg, 10 mg/kg	TLR2, TLR4, NF-κB	Parkinson’s disease (PD)	Anti-inflammatory, ROS scavenging, neuroprotection	(MPTP-induced PD model in rats)	[307]

## Data Availability

Data are contained within the article.
